# SABER-BIM: A Component-Level Adaptive Lightweighting Framework for Digital Twin BIM Models

**DOI:** 10.3390/s26102990

**Published:** 2026-05-09

**Authors:** Zhengbing Yang, Mahemujiang Aihemaiti, Beilikezi Abudureheman, Hongfei Tao

**Affiliations:** 1 College of Water Resources and Civil Engineering, Xinjiang Agricultural University, Urumqi 830052, China; xjnyyzb@outlook.com (Z.Y.); xjaubei@outlook.com (B.A.); taohongfei@xjnydx.wecom.work (H.T.); 2 Xinjiang Key Laboratory of Hydraulic Engineering Security and Water Disasters Prevention, Urumqi 830052, China

**Keywords:** digital twin, BIM lightweighting, Industry Foundation Classes (IFC), face-budget prediction, semantic awareness, mesh simplification, quadric error metrics (QEM)

## Abstract

Lightweighting Building Information Modeling (BIM) models for digital-twin applications requires balancing aggressive geometric reduction with component-level engineering tolerances and mesh usability. Most geometric simplification pipelines apply uniform ratios or hand-tuned heuristics, which struggle to accommodate the strong heterogeneity of BIM components in functional role, geometric complexity, and detail distribution. End-to-end learning-based simplification can be adaptive, but it often entangles decision-making with geometric editing, making engineering constraints difficult to enforce and audit. We present Semantic-Geometric Co-driven Adaptive Budget Estimation and Reduction for BIM (SABER-BIM), which formulates lightweighting as a component-level face-budget allocation problem. Conditioned on Industry Foundation Classes (IFC) types and structure-sensitive geometric descriptors, SABER-BIM predicts target face counts for individual components and then meets a user-specified global budget through global scaling. The predicted budgets are executed by a robust geometric backend (e.g., quadric error metrics, QEM), yielding an auditable and easily deployable pipeline. To address the absence of direct supervision, we introduce an offline pseudo-ground-truth procedure that searches for the minimum feasible target face count for each component under semantic-aware tolerance and mesh-validity constraints. Experiments on the IFCNet dataset show that SABER-BIM allocates budgets more effectively under identical global constraints, improving stability in both geometric error control and engineering usability.

## 1. Introduction

Digital twin technology has been widely adopted in industrial manufacturing, urban infrastructure, and intelligent operation and maintenance (O&M) scenarios [1,2]. When BIM models are deployed on web platforms and lightweight clients, their geometric complexity directly affects system responsiveness, rendering stability, and deployment cost [3,4]. In contrast, design-stage 3D models are created to maximize engineering fidelity and information completeness, and thus often include dense geometric detail and substantial redundancy [5,6]. This gap between design- and operation-stage objectives turns geometric complexity from a representation-accuracy issue into a practical constraint on computing, transmission, and rendering resources. Accordingly, under digital-twin operating conditions, geometric complexity should be treated as a controllable variable: redundant detail should be removed wherever possible while preserving engineering usability.

As the core data standard for BIM, Industry Foundation Classes (IFC) provides an open, vendor-neutral specification that ensures model interoperability across software platforms and throughout the building lifecycle [7]. While IFC ensures semantic consistency, its geometric representation is typically realized as a large collection of meshes composed of semantically heterogeneous components. As a result, IFC-derived meshes can impose substantial computation, transmission, and rendering costs during operation [8,9].

Mesh simplification is a standard approach for reducing geometric complexity. Among existing techniques, quadric error metrics (QEM) [10] remains popular in engineering practice because it is efficient and straightforward to implement. However, conventional pipelines typically apply a globally uniform reduction ratio or fixed heuristic parameters, which are poorly suited to BIM-based digital twin scenarios. BIM components differ fundamentally in structural role, engineering semantics, and geometric sensitivity; a one-size-fits-all strategy therefore tends to over-simplify critical components while under-compressing less important ones [11,12,13]. The key challenge is thus not the sophistication of the simplification operator, but how geometric complexity is allocated across components. While end-to-end learning methods offer adaptability, they typically entangle decision-making with geometric execution. Consequently, critical parameters—such as simplification strength and constraint enforcement—cannot be easily isolated as explicit variables, making the process difficult to audit.

**Figure 1:**
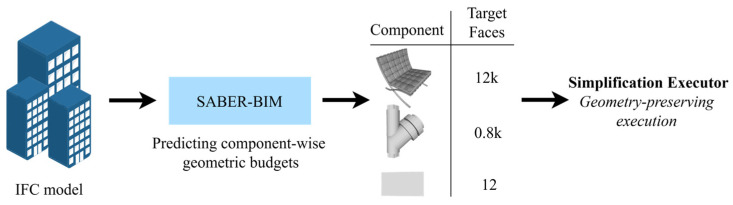
Overview of the SABER-BIM workflow. For a given IFC model, SABER-BIM predicts the minimum feasible target face count for each component, which is then enforced by a geometric simplification executor.

Building on these observations, we reformulate mesh lightweighting for BIM-based digital twins as a deterministic, component-level budget prediction problem. Specifically, for each component, we aim to predict the minimum target face count that satisfies engineering tolerances while preserving key geometric features, thereby enabling differentiated compression across components. To this end, we propose SABER-BIM, which decouples the simplification pipeline into two stages: budget prediction and geometric execution. By integrating component semantics with structure-aware geometric cues, SABER-BIM retains the stability and controllability of mature geometric simplification backends. An overview of the workflow is provided in Figure 1. The main contributions of this work are as follows:

(1) We formulate BIM lightweighting for digital-twin deployment as a component-level budget decision problem and use the target face count as an explicit decision variable to enable verifiable resource allocation.
(2) To address the lack of supervision, we develop an offline pseudo-ground-truth generation pipeline that searches, under semantic-aware tolerance and mesh-validity constraints, for the minimum feasible target face count of each component, providing reproducible supervision for budget learning.
(3) We propose a budget prediction model that integrates IFC component semantics with structure-aware geometric representations, enabling on-demand, component-wise allocation of simplification strength.
(4) We introduce a decision-execution decoupling paradigm that pairs the learning-based decision module with mature executors (e.g., QEM), improving stability, controllability, and executor replaceability in engineering deployment.

## 2. Related Work

### 2.1. Geometry-Driven Mesh Simplification Methods

Mesh simplification is a long-standing problem in geometric processing, aiming to reduce model complexity while bounding geometric error. Early work explored a range of strategies, including vertex clustering, resampling, and edge collapse operations [14,15,16]. Among these, edge-collapse-based methods have become the dominant paradigm due to their strong topological robustness and geometric stability. The quadric error metrics (QEM) method introduced by Garland and colleagues [10,16] constructs local quadric error functions to efficiently estimate the deviation induced by candidate collapse operations. By providing an effective accuracy-efficiency trade-off, QEM has become a de facto standard and is widely integrated into modeling and visualization systems [17].

Building on the QEM framework, subsequent studies have extended the original formulation by refining error metrics, introducing feature-preserving constraints, and developing adaptive weighting strategies to mitigate over-simplification in high-curvature regions and around salient geometric features [18,19,20]. For instance, Lan et al. [12] propose a saliency-preserving mechanism that improves geometric fidelity in feature-dominant areas. However, as analyzed in Section 1, these geometry-driven decisions implicitly assume model homogeneity, leading to limited sensitivity toward engineering semantics.

### 2.2. Semantic- and Rule-Driven Simplification in BIM Scenarios

Given that BIM models feature explicit component semantics and pronounced geometric heterogeneity, several studies have explored incorporating semantic information or rule-based constraints into the simplification process. For example, Zhu et al. [9] propose an IFC model simplification method guided by component semantics, where simplification strength is controlled by distinguishing component types to preserve semantic consistency in engineering contexts. Xia et al. [13] introduce multi-scale semantic information into city-scale real-scene meshes, enabling differentiated simplification across object categories. In engineering practice, a more common approach is to predefine simplification parameters or levels of detail for different components according to IFC types, functional importance, or design stages [5,6]. Such semantic- or rule-driven methods partially address the limitations of purely geometry-driven simplification by accounting for object-level differences and offer practical advantages, including straightforward implementation and explicit parameter control. However, when the model scale, instance-specific characteristics, or application requirements change, these rule parameters often require manual re-tuning, which limits adaptability to the dynamic and heterogeneous nature of BIM-based digital twin scenarios.

### 2.3. Deep Learning for Geometric Simplification

#### 2.3.1. General 3D Geometric Feature Learning Methods

With the rapid progress of deep learning, feature learning for unstructured 3D data has become increasingly mature. Representative works include PointNet and its extension PointNet++, proposed by Qi et al. [21,22]. These methods apply symmetric aggregation operators to unordered point sets, enabling unified modeling of global and local 3D features and providing a general representation framework for irregularly sampled and order-invariant geometric data. Building on this line of research, DGCNN (Dynamic Graph CNN), introduced by Wang et al. [23], employs dynamic graph convolutions to explicitly model local neighborhood relationships among points in the feature space, further enhancing the representation capacity for local geometric structures and morphological variations. These methods have achieved consistent performance in recognition tasks such as 3D object classification and semantic segmentation, and have become fundamental building blocks for learning-based 3D geometric analysis [24,25].

It is worth noting that these general-purpose 3D feature learning methods are primarily designed for recognizing and representing geometric or semantic attributes, and their typical outputs are discrete class labels or per-point semantic predictions. In contrast, mesh simplification in BIM-based digital twin scenarios requires continuous modeling and control of simplification strength, i.e., predicting simplification decision parameters rather than classifying object categories or producing semantic masks. Accordingly, in this work, such models are best viewed as encoders for geometric and structural features, extracting stable and transferable component-level representations, while the simplification decision itself must still be explicitly modeled on top of these representations.

#### 2.3.2. Learning-Enhanced Geometric Simplification and Sampling Methods

Existing studies on learning-enhanced geometric simplification do not typically follow a unified problem formulation. Instead, they intervene at different stages of the simplification pipeline, complementing or replacing components of conventional geometric simplification. According to where the learning model is incorporated, these methods can be broadly categorized into three levels:

At the local feature modeling level, learning is used to estimate the importance of local geometric features to guide the simplification operator or enhance feature preservation, e.g., saliency- or attention-based feature-aware approaches [26,27,28]. Such methods typically assign higher weights, during feature learning or error evaluation, to regions with high curvature, boundary structures, or pronounced shape variations, thereby improving the preservation of key geometric features in the simplified results.

At the geometric execution replacement level, learning models directly intervene in the execution mechanism of simplification, replacing traditional pipelines dominated by geometric error metrics with data-driven counterparts. Depending on how learning is integrated into execution, this direction can be summarized into two routes: operator-level replacement and representation-level reconstruction. Operator-level replacement trains networks to learn discrete operations or element selection during simplification. Examples include learning edge-collapse priorities in the collapse process (MeshCNN [29]), or adopting differentiable sampling strategies to perform one-shot simplification, thereby avoiding the high computational overhead of iterative collapses [30]. Similar ideas have also been extended to other geometric representations, such as point cloud sampling (S-NET [31], SampleNet [32]) and retaining or removing tetrahedral elements in volumetric meshes (TetSimNet [33]). In contrast, representation-level reconstruction does not explicitly learn an operation sequence. Instead, it learns a parameterized geometric representation with clear geometric meaning, and achieves simplification through compression and reconstruction during merging or pooling. For example, PoNQ [34] learns a joint point-normal-QEM representation and combines it with standard geometric derivations to generate meshes, where the simplification benefit mainly stems from the compressibility of the learned representation.

At the simplification decision support level, learning models are introduced to assist the simplification process while retaining the conventional geometric execution pipeline. In this setting, the model predicts simplification-related decision variables (e.g., a global retention ratio), and the actual operations are carried out by an independent geometric simplification executor. Research along this direction remains relatively limited, with representative studies mainly focusing on global parameter prediction. For example, ASimp proposed by Lin et al. [35] predicts a relative retention ratio from geometric and perceptual features to improve visual quality.

Although the above approaches introduce learning mechanisms at different stages of the pipeline, their limitations are also evident. Local feature modeling methods mainly provide a relative characterization of geometric importance, yet they do not establish an explicit mapping from feature importance to simplification strength. Geometric execution replacement methods often tightly couple simplification decisions with geometric execution, where simplification strength and performance constraints are encoded implicitly, making it difficult to obtain controllable and interpretable decision variables. While decision-support methods conceptually decouple decision-making from execution, their prediction targets are still confined to global ratios: simplification strength is specified as a relative parameter, lacking deterministic constraints that directly correspond to concrete geometric complexity. As a result, these methods often fail to provide stable and verifiable decision bases within engineering workflows.

### 2.4. Summary

In summary, existing research on mesh simplification has extended conventional pipelines from multiple perspectives, including geometric error modeling, the introduction of semantic rules, and learning-enhanced techniques. While efficient, geometry-driven methods struggle to incorporate the instance-level functional priorities required for complex BIM environments. Semantic- and rule-driven methods introduce component-level distinctions to some extent, yet their simplification strength often depends on manually designed heuristics or static configurations, and thus cannot quantitatively model the actual geometric needs of individual component instances. Learning-enhanced methods intervene in the pipeline through feature modeling, execution replacement, or decision support, improving feature awareness or automation; nevertheless, simplification strength is frequently produced as an implicit outcome or a relative ratio, lacking an explicit and verifiable decision variable that directly corresponds to geometric complexity.

In BIM-based digital twin scenarios, a model consists of numerous components with explicit semantics but highly heterogeneous geometry. Simplification therefore needs to satisfy not only global performance constraints, but also a component-level requirement: under engineering usability guarantees, how much geometric information must each component minimally retain? Existing methods have not systematically modeled this problem. In particular, there remains a lack of a unified framework that can explicitly predict target geometric complexity at the component scale and integrate this prediction with a stable geometric simplification execution process.

Accordingly, we reformulate BIM mesh lightweighting as a deterministic, component-level budget prediction problem. To guarantee engineering usability and preserve key features, the model learns to predict the required target face count for each individual component. It then derives the corresponding retention ratios under global constraints.

## 3. Model

This section presents the design and implementation of the SABER-BIM framework, a component-level adaptive mesh lightweighting approach tailored to BIM-based digital twin scenarios. Unlike conventional simplification algorithms that iteratively collapse triangles directly at the geometric execution level, SABER-BIM decomposes lightweighting into two stages: budget decision and geometric execution. This decoupled design allows the learning module to focus on the decision problem of “how much to simplify,” avoiding the instability commonly associated with end-to-end geometric generation. Meanwhile, it ensures that the predicted target face counts are verifiable and controllable, and can be seamlessly integrated into existing engineering pipelines. The remainder of this section is organized as follows:

In Section 3.1, we formally define the component-level budget prediction task and specify $F_{i}^{\min}$ as the primary form of supervision and output. Section 3.2 provides an overview of the overall SABER-BIM framework. Section 3.3 details the supervision construction mechanism. Section 3.4 introduces the network architecture of SABER-BIM. Finally, Section 3.5 presents the design of the hybrid loss function.

### 3.1. Problem Formulation

In BIM-based digital twin scenarios, a model typically consists of numerous components with explicit semantics but markedly different geometric complexities. Consider a BIM model comprising N components. Let the original triangular mesh of the *i*-th component be denoted as $G_{i}$ with an initial face count $F_{i}$. Unlike conventional mesh simplification methods [10,18,19,20], which operate directly on the geometric execution process, we reformulate BIM mesh lightweighting as a component-level simplification budget prediction problem. Specifically, for each component $G_{i}$, we learn to predict the minimum target face count $F_{i}^{\min}$ required to satisfy engineering semantics and preserve key geometric features (Section 3.3). This value is treated as the component’s geometric simplification budget for the operational stage. The component-level retention ratio can then be derived as $r_{i}=\frac{F_{i}^{\min}}{F_{i}}$. In the remainder of this paper, we use $F_{i}^{\min}$ as the primary prediction target, while $r_{i}$ is reported only as a derived metric for statistics and presentation.

### 3.2. Framework Overview

**Figure 2:**
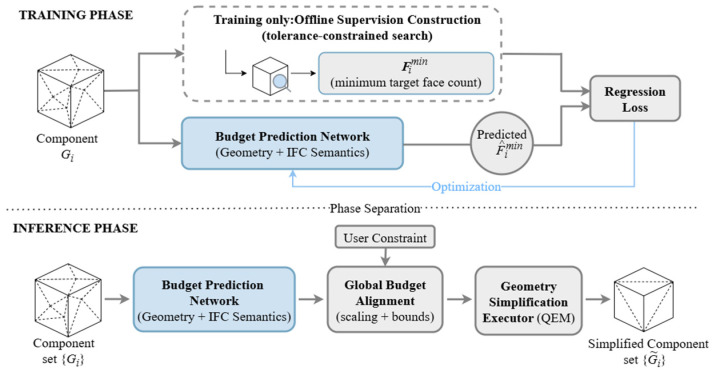
Overview of the SABER-BIM framework.

The overall SABER-BIM framework consists of two pipelines, namely the training stage and the inference stage. The training stage additionally includes a supervision construction module, whereas the inference stage retains only two steps: budget prediction and geometric execution. An overview of the framework is illustrated in Figure 2.

(1) Training stage.

During training, each individual component is treated as a basic sample. For each component mesh $G_{i}$, we first obtain its component-level minimum target face count label $F_{i}^{\min}$ through the offline supervision construction pipeline (Section 3.3). The budget prediction network then takes the component’s geometric features and IFC semantic features as inputs, and regresses the predicted ${\hat{F}}_{i}^{\min}$, which is optimized using a regression loss (Section 3.4). After training, the network learns a mapping from component features to the minimum target face count, enabling adaptive prediction of component-level budgets.

(2) Inference stage.

During inference, no offline search or ground-truth construction is performed. For each input component $G_{i}$, the budget prediction network directly outputs the predicted minimum target face count ${\hat{F}}_{i}^{\min}$. In practical deployment, users often specify a global budget, such as an overall retention ratio. To satisfy this, we apply a uniform scaling to $\left\{{\hat{F}}_{i}^{\min}\right\}$ while preserving the relative allocation trend across components. Additionally, we impose reasonable bounds to ensure the entire model strictly meets the prescribed budget. Finally, the aligned target face counts are passed as constraints to an independent geometric executor (QEM), which performs component-wise simplification and outputs the lightweight component meshes ${\tilde{G}}_{i}$.

By restricting the learning model to the budget decision layer and delegating geometric execution to a stable conventional simplification algorithm, SABER-BIM leverages this decoupled design to achieve adaptive component-level budget allocation while preserving the stability of geometric execution.

### 3.3. Supervision Construction

**Figure 3:**
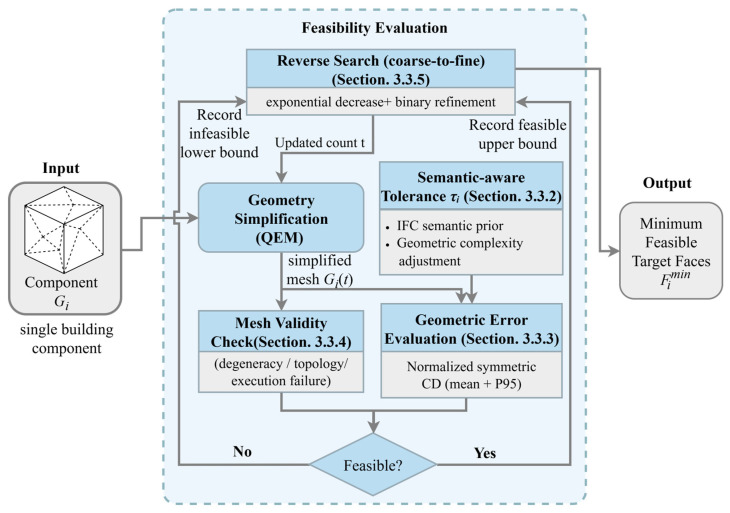
Supervision construction pipeline. Candidate target face counts are generated via a reverse search strategy. Each candidate simplification result is then evaluated for feasibility under geometric error, semantic-aware tolerance, and mesh validity constraints, thereby determining the minimum feasible target face count $F_{i}^{\min}$ for each component.

In this study, the supervision signal is the minimum target face count $F_{i}^{\min}$ to which a component $G_{i}$ can be simplified while still satisfying engineering semantics and preserving key geometric features. The minimum target face count (i.e., the optimal simplification level) lacks a directly observable, objective annotation criterion. Consequently, manually specified simplification levels often vary substantially even for the exact same component. This subjectivity makes it difficult to establish a reproducible quantitative standard. We therefore generate $F_{i}^{\min}$ offline via an automated pipeline before training and use it as the regression target for the budget prediction network. The supervision construction pipeline is illustrated in Figure 3.

To ensure clarity and reproducibility, Section 3.3 is organized as follows. Section 3.3.1 provides the label definition and the feasibility assessment framework, i.e., under what conditions a candidate target face count *t* is considered feasible. Section 3.3.2 defines how the semantic-aware tolerance threshold $\tau_{i}$ is constructed, so that the error constraints across different components share consistent engineering meaning. Section 3.3.3 specifies the geometric error metrics and the scale normalization scheme used to enable a comparable assessment against $\tau_{i}$. Section 3.3.4 describes the mesh validity checks to prevent unusable meshes from being incorrectly treated as extreme compression results. Finally, Section 3.3.5 presents the search strategy, which combines coarse and fine localization to efficiently determine the minimum feasible target face count $F_{i}^{\min}$.

#### 3.3.1. Label Definition and Feasibility Assessment

Given a component mesh $G_{i}$, we feed it into a geometric simplification executor (QEM in this study). For any candidate target face count $t\;\in \;\left[F_{i}^{low},F_{i}\right]$, we perform simplification and obtain the output mesh ${\tilde{G}}_{i}\left(t\right)$, where $F_{i}^{low}$ denotes the lower bound on the face count. A candidate t is considered feasible only if it simultaneously satisfies the following two conditions:

(1) Geometric error constraint: the geometric error of ${\tilde{G}}_{i}\left(t\right)$ relative to $G_{i}\;$ does not exceed the component-specific tolerance threshold $\tau_{i}$.
(2) Mesh validity constraint: ${\tilde{G}}_{i}\left(t\right)$ passes basic validity checks (e.g., no severe degeneracy or unusable artifacts).

Accordingly, the component-level minimum target face count $F_{i}^{\min}$ is defined as the smallest candidate face count t that satisfies the above feasibility conditions.

#### 3.3.2. Semantic-Aware Tolerance Modeling

Different components do not share the same acceptable range of geometric error. Using a uniform threshold would therefore make the resulting labels inconsistent in engineering meaning across components. To address this issue, we introduce a semantic-aware tolerance threshold $\tau_{i}$ for each component, which is determined by two factors:

Semantic prior term: the IFC component type provides a baseline tolerance, where structurally critical components are assigned stricter thresholds, while non-critical or highly redundant components are assigned more permissive thresholds. Table 1 presents the tolerance setpoints for representative building component types across IFC categories.

Geometric complexity adjustment term: the baseline tolerance is further adjusted using geometric complexity indicators, so as to prevent high-curvature or boundary-dense regions from being overly weakened during simplification.

This design does not aim to achieve an absolutely optimal numeric threshold. Instead, it defines error tolerance on a consistent scale across components, ensuring that $F_{i}^{\min}$ carries a stable engineering meaning and providing a coherent supervision scale for model learning.

#### 3.3.3. Geometric Error Metrics and Scale Normalization

To determine whether a candidate simplified result ${\tilde{G}}_{i}\left(t\right)$ satisfies the geometric error constraint, we sample points on the surfaces of the original mesh $G_{i}$ and the simplified mesh ${\tilde{G}}_{i}\left(t\right)$, and compute the symmetric Chamfer distance (CD) as the error metric. Given that local sharp structures in engineering models are particularly prone to over-simplification, we constrain both the overall error level and the tail-risk of errors: the mean error reflects global deviation, while a high-percentile error (e.g., P95) is used to suppress feature collapse caused by local outliers.

To eliminate the influence of component-scale differences on the error magnitude, we apply scale normalization during feasibility assessment, so that the tolerance threshold $\tau_{i}$ has a consistent meaning across components, thereby improving the model’s generalization capability.

#### 3.3.4. Mesh Validity Constraint and Checks

Even when the geometric error satisfies the threshold, the simplified output may still be unusable in engineering workflows due to geometric degeneracy or topological anomalies. We therefore perform basic mesh validity checks on ${\tilde{G}}_{i}\left(t\right)$, and deem a candidate infeasible under any of the following conditions:

(1) Geometric degeneracy: For example, zero-area triangles, duplicate faces, or severe collapses;
(2) Topological anomalies: For example, pronounced non-manifold edges/vertices that render the mesh unusable;
(3) Execution failure: The executor fails to produce a valid output or returns an empty result.

This constraint ensures that the resulting $F_{i}^{\min}$ corresponds to a simplified mesh with basic usability, preventing extreme compression of invalid meshes from being mistakenly treated as the minimum budget.

#### 3.3.5. Reverse Search for the Minimum Target Face Count

A direct linear scan over all candidate face counts would incur substantial offline cost. Since larger target face counts are more likely to satisfy the error and validity constraints, we adopt a two-stage reverse search strategy to locate the minimum feasible target face count:

(1) Exponential decay stage: starting from a relatively high face count, we decrease the candidate face count by a fixed ratio until the first infeasible point is encountered, thereby obtaining a feasible upper bound and an infeasible lower bound.
(2) Binary refinement stage: we perform binary search within this interval and iteratively converge to the minimum feasible target face count $F_{i}^{\min}$.

This strategy preserves the minimality of the label while significantly reducing the offline generation cost, making it suitable for supervision construction over large-scale component collections.

### 3.4. SABER-BIM Network Architecture

**Figure 4:**
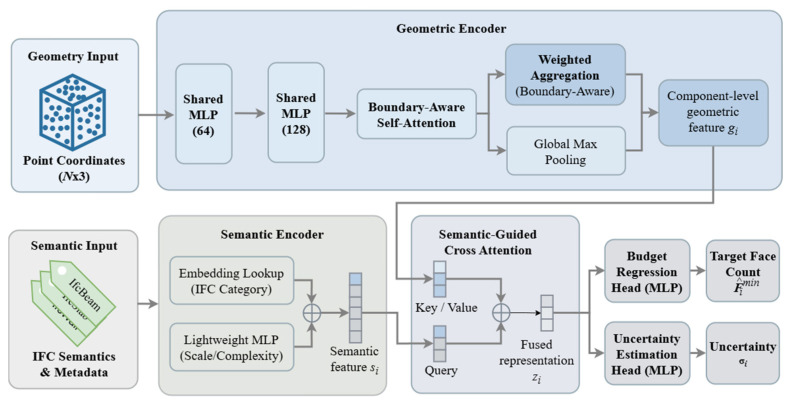
SABER-BIM network architecture.

The SABER-BIM network is built upon a semantic-aware dual-stream collaborative design. It jointly models the influences of geometric and semantic information at the component scale, with the goal of simultaneously accounting for geometric preservation and engineering semantic constraints. As illustrated in Figure 4, the overall architecture consists of four main modules: a geometric encoder, a semantic feature encoder, a cross-stream fusion module, and a multi-task prediction head. In the geometric stream, a boundary-aware attention module strengthens the representation of simplification-sensitive regions. The fused features, guided by semantics, are then used to regress the target face count and estimate predictive uncertainty.

#### 3.4.1. Geometric Encoder

To obtain component-level geometric representations, SABER-BIM adopts a lightweight point-cloud encoder that maintains computational efficiency while capturing both local details and global structural information. This design is inspired by the “point-wise feature extraction followed by global aggregation” paradigm of PointNet++ [22], yet avoids the additional overhead introduced by neighborhood sampling and ball-query operations. Instead, we combine shared MLPs (Multi-Layer Perceptrons) [21] with a self-attention mechanism [36] to more efficiently model long-range dependencies within the point set. Specifically, the encoder first applies two layers of shared 1 × 1 convolutions to map input point coordinates into a high-dimensional feature space, producing local geometric feature descriptors. It then introduces a multi-head self-attention module to capture long-range dependencies among points, particularly benefiting the representation of geometric cues in complex boundaries and high-curvature regions.

Given that mesh simplification is particularly sensitive to boundaries and sharp structures, we further introduce a boundary-aware self-supervised attention mechanism. Specifically, the encoder predicts a per-point structural sensitivity score, which is used to perform weighted aggregation of point-wise features, so that simplification-sensitive regions contribute explicitly to the component-level representation. Meanwhile, we retain a max-pooling branch to preserve strong responses to salient local features. The final component-level global geometric representation $g_{i}$ is formed by concatenating the weighted-aggregation feature with the max- pooled feature, and is further enhanced by a multi-scale feature pyramid module to improve structural expressiveness under different receptive fields.

#### 3.4.2. Semantic Feature Encoder

Relying solely on point-cloud geometry makes it difficult to reliably distinguish components that are geometrically similar but play different engineering roles, and it also fails to capture differences in physical scale across components. We therefore design an enhanced semantic feature module that incorporates the IFC component category as an explicit prior and combines it with a small set of budget-related metadata, jointly supporting component-level budget decisions.

Specifically, for the discrete IFC category labels, we employ a learnable embedding layer to map them into low-dimensional dense vectors, capturing latent differences in simplification requirements across component types. Meanwhile, since even components of the same type may demand different budgets under varying physical scales and initial complexities, the semantic encoder can further incorporate budget-related metadata (e.g., component scale/complexity) and encode them into scale-aware features via a lightweight MLP. The resulting features are concatenated along the channel dimension to form an integrated semantic representation $s_{i}$, which serves as an input to the subsequent semantic-geometric fusion module.

It is important to emphasize that $s_{i}$ is not used to instantiate handcrafted rules. Instead, it serves as a learnable semantic prior in feature fusion, allowing the network to adaptively adjust budget allocation strategies for components of different categories and scales during end-to-end training. This design improves sensitivity to engineering semantic differences and enhances cross-scenario generalization.

#### 3.4.3. Semantic-Guided Cross-Feature Fusion

After obtaining the multi-scale geometric representation $g_{i}$ and the semantic representation $s_{i}$ for each component, a straightforward approach is to concatenate them and perform regression. However, semantic information is low-dimensional and acts as a discrete prior. Under naive concatenation, this semantic signal is easily diluted by high-dimensional geometric features. Such dilution makes it difficult to explicitly modulate the network’s attention across different geometric channels and structural patterns. To address this issue, SABER-BIM introduces a semantic-guided cross-attention fusion module, where semantics serve as a conditioning signal and attention is used to selectively aggregate geometric features. This produces a more discriminative joint representation for budget regression.

Specifically, we use the semantic feature as the query (*Q*) and the geometric feature as the key (*K*) and value (*V*). We compute semantic-geometric association weights via scaled dot-product attention and obtain the fused feature $f_{i}$ by weighted aggregation over the geometric features:

$$f_{i}=Attn\left(s_{i},g_{i}\right)\;=Softmax\left(\frac{\left(W_{Q}s_{i}\right){\left(W_{K}g_{i}\right)}^{T}}{\sqrt{d}}\right)\left(W_{V}g_{i}\right)$$

where $W_{Q}\in {\mathbb{R}}^{d\times d_{s}}$, $W_{K}\in {\mathbb{R}}^{d\times d_{g}}$, and $W_{V}\in {\mathbb{R}}^{d\times d_{g}}$ are learnable weight matrices, and *d* denotes the feature dimension. Here, $d_{s}$ and $d_{g}$ represent the dimensions of the input semantic features and geometric features, respectively.

This fusion scheme allows semantic information to participate in geometric representation learning in a conditioned manner: under different IFC types and scale conditions, the model can adaptively adjust its responses to geometric channels and structural regions. As a result, the semantic signal is not weakened as in naive concatenation, and the resulting joint features provide a more stable and discriminative basis for regressing the minimum target face count.

#### 3.4.4. Prediction Heads and Outputs

The budget prediction head takes the fused representation $z_{i}$ as input. After extracting a task-agnostic high-level representation via shared MLP layers, it branches into a regression head and an uncertainty head, which are used to predict the minimum target face count and to quantify confidence, respectively.

Given the large dynamic range and pronounced long-tailed distribution of face counts in BIM components, the regression head outputs ${\hat{y}}_{i}$ in the log domain (corresponding to a prediction of $\log(1+{\hat{F}}_{i}^{\min})$). During inference, we apply the inverse transform to obtain ${\hat{F}}_{i}^{\min}$, and clip it to the valid interval $\left[F_{i}^{low},F_{i}\right]$, ensuring that the output can be directly used as a target face-count constraint for QEM.

To quantify prediction reliability, the uncertainty head outputs a non-negative scale parameter $\sigma_{i}$ (enforced via Softplus for numerical stability), which characterizes sample-wise predictive risk. During inference, $\sigma_{i}$ can serve as a risk indicator to provide a conservative margin for budget alignment and simplification strategies, thereby enabling more controllable simplification decisions.

### 3.5. Hybrid Loss Function

The training objective of SABER-BIM is not only to accurately regress the component-level minimum target face count $F_{i}^{\min}$, but also to maintain optimization stability over BIM components with extremely large face-count ranges and substantial geometric diversity, and to prevent feature fusion failure caused by attention degeneration. To this end, we adopt a weighted hybrid objective that, on top of log-domain regression, jointly incorporates heteroscedastic uncertainty modeling and an attention regularization term. The overall loss is defined as:

$$L_{total}=\lambda_{main}L_{main}+\lambda_{unc}L_{unc}+\lambda_{att}L_{att}\;$$

where λ denotes the weighting coefficients used during training. The three terms correspond to budget regression, uncertainty calibration, and attention regularization, respectively.

#### 3.5.1. Budget Regression Loss $L_{main}$

The primary task is to regress the component-level minimum target face count $F_{i}^{\min}$. To mitigate training instability caused by the wide dynamic range of face counts, we perform regression in the log domain. Specifically, we map the ground-truth face count to $y_{i}=\log(1+F_{i}^{\min})$, and the network outputs ${\hat{y}}_{i}$. We then use mean squared error (MSE) between ${\hat{y}}_{i}$ and $y_{i}$ as the main regression loss $L_{main}$. This log-domain formulation improves optimization stability without altering the definition of the prediction target, and it also aligns the regression space with the subsequent uncertainty modeling term.

#### 3.5.2. Uncertainty Calibration Loss $L_{unc}$

To improve robustness on complex components, the network additionally outputs a non-negative uncertainty value $\sigma_{i}$ to characterize sample-wise prediction difficulty. Intuitively, when certain components are harder to fit or their labels lie closer to the tolerance boundary, the model is allowed to produce higher uncertainty to reflect increased predictive risk. Meanwhile, uncertainty itself must be regularized to prevent the model from trivially inflating $\sigma_{i}$ to evade regression errors. We therefore calibrate $\sigma_{i}$ using a heteroscedastic negative log-likelihood (NLL) objective as the uncertainty loss $L_{unc}$, optimized jointly with the main regression term. The uncertainty calibration loss $L_{unc}$ is defined as follows:

$$L_{unc}=\frac{1}{N}\overset{N}{\underset{i=1}{\sum }}\left[\frac{{\left(\hat{y_{i}}-y_{i}\right)}^{2}}{2\sigma_{i}^{2}}+\frac{1}{2}\log\sigma_{i}^{2}\right]$$

Under the dynamic constraint of this formulation, if the model exhibits high uncertainty for a specific prediction (i.e., a large σ), the residual penalty in the first term is attenuated, thereby preventing the network from forcefully fitting difficult samples. Concurrently, the logarithmic regularization penalty in the second term increases to prevent the model from evading regression computation by meaninglessly inflating the variance. This design encourages the model to increase uncertainty on challenging samples rather than forcing the errors to be uniformly flattened, thereby improving overall training stability.

#### 3.5.3. Attention Regularization $L_{att}$

In the geometric encoding and semantic-guided fusion stages, attention mechanisms are employed to model long-range dependencies within the point set and to form a more discriminative joint representation. However, in the early phase of training, attention weights may degenerate into an approximately uniform distribution (lacking discriminability) or collapse into overly concentrated mass on a few uninformative positions, which weakens the modeling of key structural relationships and destabilizes budget regression. To alleviate this issue, we introduce a lightweight regularization term $L_{att}$ on the attention weights, encouraging the attention distribution to retain effective discriminability while maintaining a moderate degree of focus. The regularization term $L_{att}$ is defined as follows:

$$L_{att}={∥{AA}^{T}-I∥}_{F}^{2}$$

where $A\in {\mathbb{R}}^{h\times d}$ denotes the weight distribution matrix of the *h* attention heads, *I* is the identity matrix, and ${∥\cdot ∥}_{F}^{2}$ represents the squared Frobenius norm. This regularization constrains only the shape of the attention distribution, without prescribing specific regions that must be attended to, thereby improving the effectiveness of the fusion module while preserving model expressiveness.

## 4. Experiments and Results Analysis

### 4.1. Dataset and Experimental Setup

#### 4.1.1. Dataset

To evaluate the generalization capability of SABER-BIM in handling heterogeneous BIM components from real-world engineering scenarios, we adopted IFCNet [37] as the experimental benchmark. This dataset was derived from parsing IFC models collected from approximately 1000 practical building projects and covers a variety of common BIM geometric representations, including B-Rep, sweep, CSG, and subdivision surfaces [38]. During geometric decomposition, the core IFC semantic attributes of each component were preserved, providing the foundation for our semantic-aware simplification strategy.

In our experiments, we curated 17,368 independent IFC components, spanning typical engineering objects such as structural elements, MEP pipeline systems, and architectural/decorative components. The dataset exhibits pronounced geometric-semantic heterogeneity: component face counts vary widely (from tens to approximately 10^5^), and geometric complexity does not follow a simple correspondence with engineering semantics. This property makes it a suitable benchmark for assessing whether a method can achieve truly adaptive simplification.

To improve robustness to spatial transformations, we applied two-fold data augmentation to the base samples (e.g., random rotations and scale perturbations), yielding 34,736 valid samples in total. The dataset is split into training/validation/test sets with an 8:1:1 ratio, while strictly enforcing an object-level separation protocol: all augmented variants derived from the same original component are kept within the same subset, preventing any cross-split leakage.

#### 4.1.2. Implementation Details

To ensure the reliability and reproducibility of the experimental results, all model training and inference are conducted under a unified computing environment. SABER-BIM is implemented in PyTorch 2.9.1, and the hyperparameters are selected to balance training stability and convergence accuracy. The experimental environment and key training settings are summarized in Table 2.

Given the parameter configurations and training set scale detailed in Table 2, the average duration for a single training epoch is approximately 7 min. The total time required to complete the prescribed 100-epoch training process is roughly 13 h. Such computational costs are well within acceptable limits for the offline pre-training stage. Furthermore, during the inference phase, the trained model processes an individual component in just 12.4 ms, demonstrating its capacity to meet the high-efficiency demands of practical BIM engineering.

#### 4.1.3. Analysis of Training Dynamics

**Figure 5:**
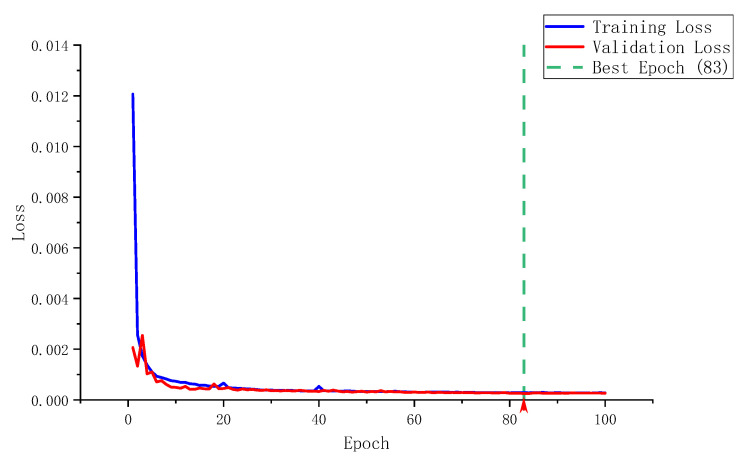
Learning curves of the SABER-BIM model over 100 training epochs. The training and validation losses converge rapidly in the initial stage, and the minimal gap between the final convergence values indicates a stable learning process with no apparent overfitting.

The dynamics of the training process serve as a crucial indicator for assessing the health and learning efficiency of the deep learning model. Figure 5 illustrates the learning curves of the training and validation losses for SABER-BIM over the 100-epoch training cycle. Additionally, Table 3 summarizes the key statistical metrics recorded during the training process.

Based on the loss curves in Figure 5 and the statistical data in Table 3, three key dynamic characteristics of the training process can be observed:

1. Rapid Convergence: During the first 20 epochs, the loss values exhibit a steep downward trajectory. This indicates that the PointNet++ based geometric encoder can swiftly capture the global geometric features of the components, allowing the model to quickly escape the random initialization state.
2. Smooth Optimization: From epoch 20 to 80, the deceleration of the loss reduction becomes apparent, yet it maintains a monotonically decreasing trend. This is primarily attributed to the intervention of the ReduceLROnPlateau learning rate scheduling strategy (triggered 5 times in total), which enables the model to perform refined searches near local minima, effectively preventing oscillations during the later stages of training.
3. Excellent Generalization: The most prominent feature throughout the training process is the remarkably small gap between the training and validation losses. As shown in Table 3, the difference between the final training loss (0.273 × 10^−3^) and the validation loss (0.260 × 10^−3^) is a mere 0.013 × 10^−3^. These nearly overlapping curves strongly validate the architectural rationality of the SABER-BIM model. Furthermore, these results demonstrate that Dropout regularization and data augmentation effectively suppress overfitting. Consequently, the model learns generalizable geometric and semantic representations instead of merely memorizing the training data.

#### 4.1.4. Quantitative Evaluation on the Test Set and Main Results

To quantify the generalization performance of SABER-BIM on unseen components, we conducted a comprehensive evaluation of regression accuracy using the trained model on an independent test set comprising 1992 samples. The evaluation metrics encompass Mean Absolute Error (MAE), Root Mean Square Error (RMSE), and the Coefficient of Determination ($R^{2}$). Additionally, the Mean Absolute Percentage Error (MAPE) was introduced to measure prediction deviation at the percentage level. Table 4 details the core performance metrics on the test set. The results indicate that SABER-BIM exhibits exceptionally high regression fidelity in the geometric simplification rate prediction task.

The data demonstrate that the model achieves an MAE of 0.066 on the normalized budget (retention ratio). From a practical engineering perspective, this implies that if the true optimal retention ratio of a component is 0.70, the model’s prediction will reliably fall within the narrow interval of [0.634, 0.766]. Given that BIM components frequently consist of tens of thousands of faces, this precision provides a highly accurate simplification prior for engineering applications. Furthermore, the high coefficient of determination ($R^{2}=\;0.89$) confirms that the variance in the model’s predictions highly explains the variance in the ground truth labels. This proves that the overall regression trend of SABER-BIM is highly consistent with real-world engineering demands and is not severely perturbed by individual extreme or geometrically complex samples.

### 4.2. Validating the Reasonableness of the Pseudo Ground Truth

Since the component-level supervision signals in this work are automatically generated by the semantic-aware reverse optimization procedure described in Section 3.3, it is necessary to first verify their reasonableness under engineering tolerance constraints. We randomly sampled 100 IFC components from the test set and constructed three settings based on the ground-truth retention ratio: GT, GT − 10%, and GT + 10%. Under a unified error measurement protocol and a consistent definition of the relative tolerance threshold, we report relative geometric error metrics alongside the tolerance violation rate (TVR)—the proportion of samples exceeding this threshold. These statistics are used to assess whether GT lies near the empirical boundary corresponding to the minimum retention ratio that still satisfies the tolerance constraints. The results are summarized in Table 5.

As shown in Table 5, compared with GT, over-simplification (GT − 10%) leads to a pronounced increase in TVR (from 2% to 15%), while both the *P*95 and the mean of the relative Chamfer distance increase simultaneously. This indicates that further tightening the budget causes more components to exceed the tolerance threshold and introduces larger overall geometric deviations. In contrast, under-simplification (GT + 10%) continues to reduce the relative distance errors, yet TVR does not decrease further (remaining at 2%), suggesting that performance has entered a regime of diminishing returns under the current tolerance threshold.

It should be noted that GT ± 10% is constructed by perturbing the ground-truth retention ratio. In implementation, the target face count is subject to upper/lower clipping (no larger than the original face count and no smaller than a prescribed minimum) and integer rounding. Consequently, for some components, the perturbation yields only limited changes in the actually attainable face counts, which explains the relatively small change in the average retention ratio for GT + 10% in Table 5. Taken together, these observations show that GT achieves a more appropriate trade-off between suppressing tolerance violations and controlling geometric errors, and lies close to the empirical feasibility boundary induced by the tolerance constraints. Therefore, it serves as an effective supervision signal for the budget prediction task.

### 4.3. Comparative Experiments

To evaluate the budget allocation capability of SABER-BIM for lightweighting IFC components in digital twin scenarios, we compare it, under a unified evaluation protocol, against fixed-ratio QEM and representative learning-based budget prediction methods. All experiments follow the same two-stage paradigm of budget decision and geometric execution: each method is treated as a component-level budget decision maker (outputting a target face count or an equivalent retention ratio), and its outputs are subsequently fed into the same QEM pipeline to perform simplification. This design attributes performance differences primarily to budget allocation strategies, rather than to the underlying simplification operator.

It is worth noting that learning-based methods that are consistent with our setting, i.e., those that can directly output component-level budgets and be integrated into the above alignment protocol remain relatively limited. Among them, ASimp [35] is the closest to our formulation and is therefore selected as the main learning-based baseline. We also evaluated end-to-end mesh simplification methods such as NMS (Neural Mesh Simplification) [30] and PoNQ [34] to examine their engineering suitability on BIM components. However, these approaches directly generate simplified meshes without exposing an explicit budget interface, making them difficult to incorporate into the budget-alignment protocol for fair comparison. Moreover, they are more prone to producing unusable geometries (e.g., non-manifold artifacts or degenerate faces) on BIM component meshes, which substantially increases distance errors and invalid-mesh rates. Accordingly, our primary conclusions are drawn from comparable comparisons among budget decision-makers, while end-to-end methods are reported only for reference and are not included in the main tables. All models are evaluated on the same augmented IFCNet test set, using the best checkpoint selected on the validation set.

#### 4.3.1. Budget Efficiency and Decision Accuracy

**Figure 6:**
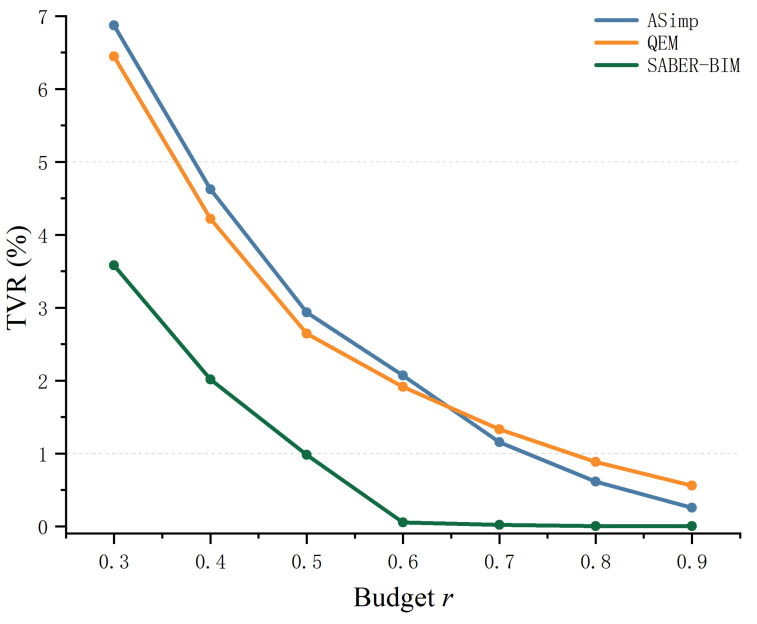
TVR-budget retention ratio curves.

**Figure 7:**
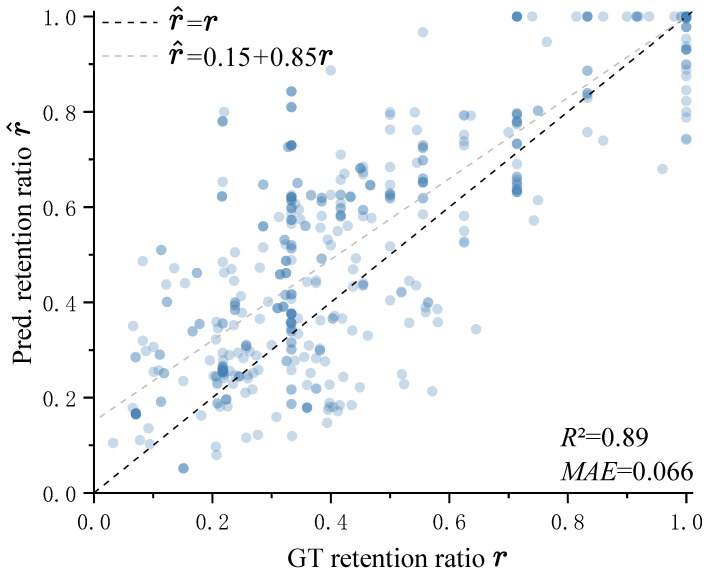
Calibration scatter plot.

The value of budget prediction lies in whether it can allocate faces effectively under tolerance constraints. We analyze budget prediction from two perspectives: budget efficiency and decision accuracy. Specifically, we use the TVR-budget retention ratio curve to characterize tolerance feasibility across different global budgets and compare the minimum budget required to reach a target TVR (Figure 6). We further examine the calibration relationship between the predicted and ground-truth retention ratios to assess the reliability of component-level allocation and potential systematic bias (Figure 7).

As shown in Figure 6, SABER-BIM achieves lower TVR across all budget levels, indicating that it is more likely to satisfy component tolerances under the same face-count constraints. Moreover, when fixing a TVR threshold (e.g., 1% or 5%), the intersection between SABER-BIM and the threshold line shifts further left, meaning that it attains the same tolerance feasibility with a smaller global retention ratio, which reflects higher budget utilization efficiency.

Figure 7 presents a calibration scatter plot between the predicted retention ratio and the ground-truth retention ratio. The samples largely align with the diagonal with small deviations (as reflected by the reported *R*^2^ and MAE), suggesting that the model can accurately estimate the retention level required for component-wise tolerance feasibility. The linear fit, $\hat{r}=0.15+0.85r$, indicates an approximately linear agreement between prediction and ground truth: the slope being close to 1 implies limited global scaling bias, while the positive intercept corresponds to a mildly conservative tendency in the low-retention regime. Such conservatism helps reduce under-allocation risks caused by over-compression. At the global level, this component-wise calibration manifests as the lower TVR and smaller “meeting-the-threshold” budgets observed in Figure 6.

#### 4.3.2. Quantitative Comparison

**Figure 8:**
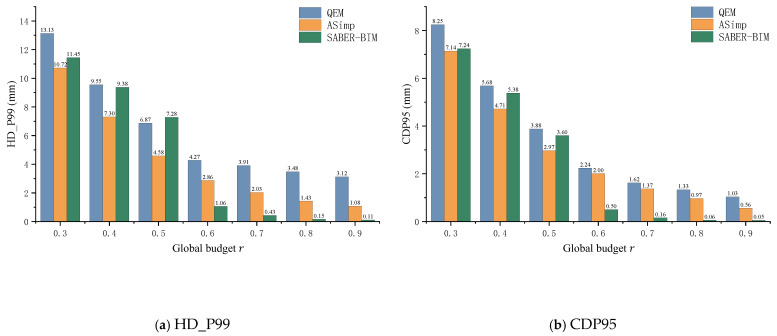
Geometric error comparison under different global budgets: (**a**) HD_P99 and (**b**) CDP95. Note: HD_P99 (99th Percentile Hausdorff Distance) quantifies the maximum local deviation between the original and simplified meshes. It is highly sensitive to local feature collapse or boundary distortion, serving as a core metric for evaluating the worst-case usability of engineering models. CDP95 (95th Percentile Chamfer Distance) reflects the overall geometric stability after filtering out extreme outliers. For all metrics, lower values indicate smaller errors (↓).

Since the component-level budget face counts (${\hat{F}}_{min}$) predicted by different learning-based methods vary, we established a unified global budget constraint and a budget-alignment protocol to ensure a strictly fair comparison under identical geometric resource overheads. Specifically, the global budget constraint mandates that all comparative methods must achieve an identical final global retention ratio (e.g., $r\;\in \left\{0.9,0.7,0.5,0.3\right\}$). To achieve this, the budget-alignment protocol performs a unified linear scaling on the predicted face counts to strictly align them with the global budget. Table 6 reports retention statistics before and after alignment. The two learning-based decision makers yield final global retention ratios that are close to the target budgets at each operating point, supporting the comparability of the subsequent error and usability results. Figure 8 summarizes the trends of HD_P99 and CDP95 over the full budget range r ∈ [0.3,0.9]. Table 7 provides detailed comparisons at three representative budget levels $r\;\in \left\{0.7,0.5,0.3\right\}$. The best result in each column is highlighted in bold.

Figure 8 shows that as the budget becomes tighter (smaller r), both HD_P99 and CDP95 increase, which is consistent with the expected error accumulation under stronger simplification. Compared with QEM and ASimp, SABER-BIM achieves lower HD_P99 and CDP95 in the mid-to-high budget regime (e.g., r ≥ 0.6), indicating that its budget allocation is more effective at controlling both worst-case and overall deviations in this range. Under more aggressive compression (r ≤ 0.5), tail errors tend to be dominated by a small number of sensitive components, and the performance gaps become more pronounced and method-dependent.

For a clearer quantitative view, Table 7 summarizes results at *r* ∈ {0.7, 0.5, 0.3}, reporting tail error (HD_P99), overall errors (CDP95, CE), and engineering usability indicators (WA, Invalid). At *r* = 0.7, SABER-BIM achieves the best overall balance, with consistently low geometric errors while maintaining low WA and Invalid. At *r* = 0.5 and *r* = 0.3, SABER-BIM remains competitive in overall error (CDP95) and keeps usability-related metrics comparatively stable. Overall, SABER-BIM provides a more favorable trade-off between geometric error control and engineering usability across most budget levels, supporting the effectiveness of component-level adaptive budget allocation in BIM scenarios.

#### 4.3.3. Qualitative Comparison

**Figure 9:**
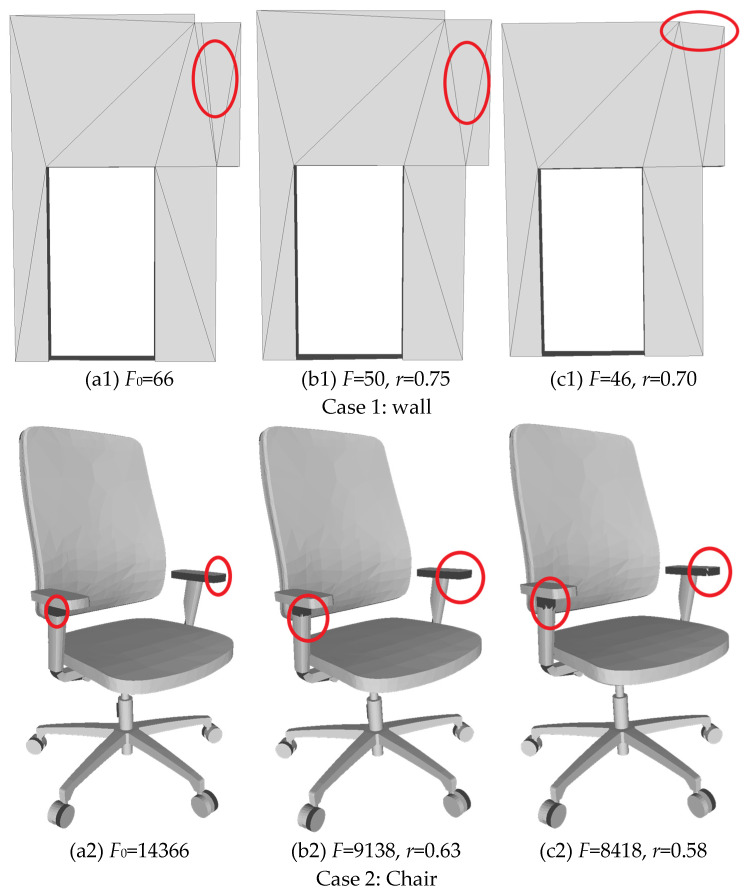

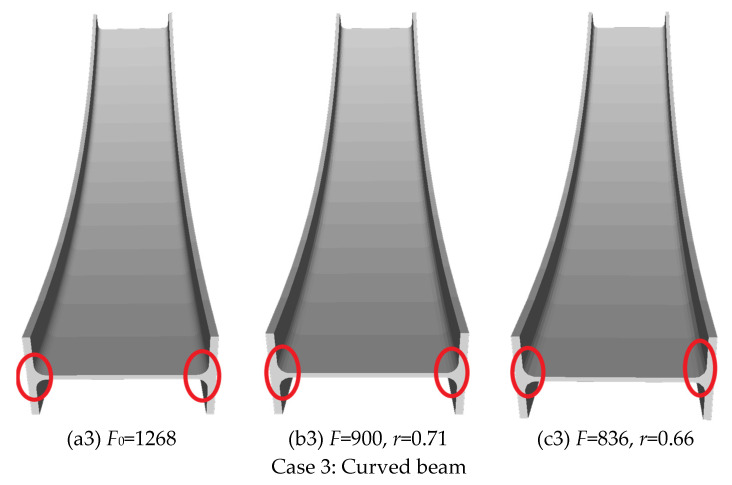
Simplification results under different retention ratios. Each row shows one representative component. The three columns correspond to: (**a1**–**a3**) the original mesh; (**b1**–**b3**) the simplification result under the predicted retention ratio $\hat{r}$ and (**c1**–**c3**) the result under a further tightened budget ($\hat{r}$ − 0.05). Red circles indicate tolerance-sensitive regions, highlighting where local degradation first emerges when the budget is tightened. The face count *F* and retention ratio *r* are reported under each subfigure.

To provide a more intuitive view of simplification behavior under tolerance constraints, we visualize three representative component categories in Figure 9: a wall with open boundaries, a complex non-structural object with thin connections (a chair), and a boundary-sensitive curved beam. These examples are used to inspect where and how local degradations are triggered under the predicted retention ratio $\hat{r}$ and under a tightened perturbation ($\hat{r}$ − 0.05), highlighting differences in the location and form of the first noticeable failures.

Across all three cases, a consistent trend can be observed. Under the predicted retention ratio $\hat{r}$, the key structural regions remain largely stable, with no obvious local collapse or shape drift. When the budget is further tightened to ($\hat{r}-0.05$), local degradation first appears at the circled sensitive regions: the wall boundary deforms, fine details around the chair armrest are weakened, and the ends of the curved beam exhibit more pronounced contour shrinkage. These observations suggest that the predicted $\hat{r}$ often lies close to the tolerance-feasibility boundary, achieving a favorable balance between compression and geometric fidelity. Further reducing the retention ratio tends to distort tolerance-sensitive regions first, thereby increasing the risk of violating tolerance constraints.

### 4.4. Ablation Study 

To quantify the contributions of individual modules to budget decisions and downstream simplification quality, we conduct an ablation evaluation under the same budget-alignment setting as in the main experiments. We consider three representative budget levels $r\in \left\{0.7,0.5,0.3\right\}$. Using the full model (baseline) as the reference, we construct four variants: mse_only, no_multi_scale, no_semantic, and no_uncertainty. Table 8 summarizes mesh quality and usability metrics under different budgets, while Table 9 reports the regression accuracy of budget prediction.

The results show that the full model maintains a more stable trade-off between quality and usability across all three budget levels. Removing the semantic branch (no_semantic) resulted in substantial degradation: the predictive fitting capability declined noticeably, and geometric errors escalated concurrently across all budget levels. This outcome not only demonstrates the critical role of semantic information in distinguishing the tolerance sensitivity of components, but the no_semantic variant also objectively simulates a worst-case scenario in real-world engineering where IFC metadata is completely missing. The data indicates that even in the extreme condition of total semantic label deprivation, the model—despite experiencing performance degradation—retains the ability to execute foundational budget allocation relying purely on geometric features. Removing the uncertainty term (no_uncertainty) also harms stability, manifested by higher prediction errors and noticeably larger geometric deviations under moderate budgets, indicating that uncertainty modeling plays an important role in risk control and in avoiding under-allocation. Removing the multi-scale pyramid (no_multi_scale) primarily affects geometric fidelity under tight budgets, highlighting the necessity of multi-scale structure for representing details and structure-sensitive regions.

To verify the reliability of the performance differences between the aforementioned ablation variants and the full model, this study further conducted statistical hypothesis testing. Based on the absolute error distribution on the test set, we calculated the 95% confidence intervals (95% CI) and employed a paired sample t-test to evaluate statistical significance. The statistical analysis indicates that although the regression accuracy of the full model (SABER-BIM Full) is numerically slightly inferior to the mse_only variant (with a MAE difference of approximately 0.003), the difference in their error distributions does not reach statistical significance (p=0.12 > 0.05). However, as shown in Table 8, mse_only exhibits a substantial degradation in actual mesh generation quality (e.g., HD_P99 and invalid mesh ratio). This confirms the core thesis of this study from both statistical and geometric perspectives. Relying solely on MSE optimization to pursue a marginal, statistically insignificant numerical advantage cannot satisfy the strict tolerance demands of practical BIM engineering.

Overall, the semantic-geometric co-modeling and uncertainty mechanism of SABER-BIM are both necessary and complementary for adaptive budget decision–making under tolerance constraints.

## 5. Discussion

### 5.1. Budget Decisions and Engineering Reliability

Lightweighting BIM models for digital twin scenarios is not merely geometric compression; rather, it is a constrained geometric resource optimization problem: under fixed storage and bandwidth budgets, the goal is to preserve as much geometric information as possible that is critical for engineering interpretation and visualization. Our experimental results (Section 4) empirically confirm that BIM’s semantic heterogeneity necessitates a conditioned lightweighting approach, moving beyond the limitations of uniform ratios. In this work, we incorporate IFC types as conditional information and jointly leverage geometric structural cues at the component-instance level for budget prediction, capturing both budget sensitivity across semantic categories and instance-level variations within the same category. This enables a more reasonable reallocation of budgets under the same global constraint (see the quantitative results in Section 4.3.2).

We further model the simplification decision as component-level target face count prediction, rather than predicting a relative simplification ratio as in ASimp [35]. The primary motivation is engineering controllability and verifiability. The mapping from a relative ratio to the final geometric scale is jointly affected by the original complexity, scale differences, and executor behavior, which can undermine consistency across components and projects. In contrast, the target face count is a decision variable with a clear physical unit, naturally aligning with global budget constraints. This makes it easier to audit and verify, and provides a clearer decomposition path for error diagnosis. When outcomes deviate from expectations, the sources of error can be separated into budget decision bias versus executor response differences, thereby reducing uncertainty in engineering deployment.

From a system-design perspective, we adopt a decoupled paradigm of “budget decision-geometric execution”, primarily for engineering reliability and controllability. By retaining a mature geometric simplification executor, the system reduces the risk of amplifying topological degeneration and mesh invalidity during learning. This decoupled design also clarifies the learning module’s responsibility boundary. Specifically, the model only needs to learn resource allocation, entirely avoiding the need to learn complex geometric editing end-to-end. This decoupling also provides executor replaceability: without changing the decision model, the execution module can be swapped according to platform or pipeline constraints, improving integration flexibility with existing engineering systems. Finally, it is worth noting that different methods often optimize different objectives under different execution assumptions. Our comparative conclusions therefore primarily reflect engineering suitability under a unified executor, budget alignment, and usability-constrained setting, and should not be interpreted as a universal ranking across tasks or scenarios.

### 5.2. Limitations and Future Work

Although the proposed method achieves stable gains under our current evaluation setting, the conclusions are still conditioned on certain engineering assumptions and data characteristics. It is therefore necessary to clarify the scope of applicability and outline directions for further validation.

(1) Consistency of IFC semantic granularity. We use IFC types as conditional information to capture budget sensitivity across components. As a result, model behavior depends to some extent on the granularity and consistency of semantic labels. For engineering data with type merging/splitting, project-specific conventions in type usage, or missing semantic fields, the semantic prior and its mapping to budgets may require recalibration. Future work will systematically evaluate robustness with respect to IFC granularity and semantic missingness/uncertainty, and investigate graceful degradation strategies when semantic conditions are unreliable.
(2) Dependence of supervision on engineering assumptions. Our supervision signals are generated offline under the joint constraints of error tolerance and mesh validity. This provides labels with clear engineering semantics at the feasibility boundary; however, the boundary location is influenced by the tolerance threshold design, the choice of error metric, and the validity-check rules. We will conduct sensitivity analyses on tolerance strength and perform rule-substitution experiments to quantify how supervision design affects the learned outcomes.
(3) Protocol boundary induced by a unified executor. We fix the geometric executor in our comparisons to control confounding factors, aiming to evaluate the engineering suitability of budget allocation strategies themselves. Therefore, the conclusions should be interpreted primarily as effectiveness under this executor and evaluation protocol, rather than as a universal ranking of end-to-end simplification approaches. Future work will examine transferability by reproducing results across different executors and conducting cross-executor consistency evaluations.
(4) Missing context under component-wise independent modeling. We treat each component as an independent decision unit for scalability and engineering integration. However, the current formulation does not explicitly leverage system-level context such as assembly relations, connectivity constraints, or cross-component continuity. In scenarios with strongly coupled structures, such context may influence global consistency. Future work will explore lightweight relationship modeling or context prompting mechanisms to improve system-level coherence without breaking the “decision-execution decoupling” framework.

## 6. Conclusions

We present SABER-BIM, a semantic-conditioned framework for component-level simplification budget prediction in BIM-based digital twin scenarios. We explicitly model the simplification decision as component-level target face count prediction and decouple it from geometric execution, resulting in a lightweighting pipeline that is verifiable, auditable, and easy to integrate into engineering workflows under global budget constraints. The proposed framework provides a reusable modeling paradigm for achieving stable budget allocation on BIM data with pronounced semantic heterogeneity and substantial instance-level variations.

Future work will extend validation along several directions, including robustness to semantic conditions, sensitivity to supervision design, cross-executor consistency, and incorporating inter-component context modeling. We will also add a tolerance-driven minimum-face-count cost evaluation to more comprehensively characterize the applicability boundary and engineering benefits of the proposed method.

## Figures and Tables

**Table 1:** Tolerance settings for representative IFC component categories.

Tolerance Group	Representative IFC Class	Base Tolerance τ*_base_* (Relative)	Description
Strict Tolerance	IfcWall	3 × 10^−3^	Primary structural components; requires strict constraints to preserve boundary accuracy.
	IfcColumn		
	IfcBeam		
Moderate Tolerance	IfcPipeSegment	1.5 × 10^−2^	Functional components; surface details can be moderately simplified provided the overall contour is preserved.
	IfcDuctSegment		
	IfcValve		
Relaxed Tolerance	IfcFurniture	3 × 10^−2^	Non-core components with high geometric redundancy; tolerances are relaxed to maximize lightweighting gains.
	IfcWindow		
	IfcRailing		

**Table 2:** Experimental environment and hyperparameter configuration.

Item	Setting
GPU	NVIDIA GeForce RTX 4060 Ti (8 GB)
CUDA/cuDNN	CUDA 12.6/cuDNN 9.x
Framework	PyTorch 2.9.1 + cu126 (Python 3.10)
Input	1024 points per component; unit-sphere normalization
Optimizer	AdamW (weight decay = 5 × 10^−5^, *β*_1_ = 0.9, *β*_2_ = 0.999)
Learning-rate schedule	initial LR = 5 × 10^−4^; ReduceLROnPlateau
Batch size	32
Training	up to 100 epochs; early stopping (patience = 10; min epochs = 20)
Dropout	0.2
Loss	Adaptive Target-Face Loss

**Table 3:** Training performance statistics.

Metric	Value	Description
Total Epochs	100	Scheduled training cycle
Best Epoch	83	Model checkpoint node
Best Validation Loss	0.260 × 10^−3^	Lowest recorded validation error
Final Training Loss	0.273 × 10^−3^	Training error at convergence
Loss Reduction	97.74% (Train)/87.83% (Val)	Optimization efficiency indicator

**Table 4:** Quantitative performance metrics on the test set.

Metric	MAE	RMSE	MAPE	*R* ^2^	Avg. Uncertainty
Value	0.066	0.129	17.86%	0.890	0.314

**Table 5:** Validation of pseudo ground-truth reasonableness.

Setting	Avg. Retention Ratio	TVR (%)	CD P95_rel_ (×10^−3^)	CD_rel_ Mean (×10^−3^)	tol_rel_ Mean (×10^−3^)
GT − 10%	0.67	15	10.93	2.16	14.0
GT	0.73	2	6.09	1.38	14.0
GT + 10%	0.79	2	5.62	1.08	14.0

Note: “Setting” denotes perturbations applied to the GT retention ratio (Δ = ±10%). Except for tol_rel_ mean, all metrics are lower-is-better.

**Table 6:** Budget alignment and executed retention statistics (SABER-BIM (Ours) vs. ASimp).

Budget	Raw Mean Retention Ratio (Ours)	Raw Mean Retention Ratio (ASimp)	Final Global Retention Ratio (Ours)	Final Global Retention Ratio (ASimp)
0.9	0.747	0.695	0.887	0.899
0.7			0.694	0.700
0.5			0.49	0.502
0.3			0.307	0.303

Note: The raw mean retention ratio is defined as the arithmetic mean of the component-level retention ratios directly output by the network without global constraints, reflecting the model’s initial estimation of the components’ simplification potential. The final global retention ratio is defined as the ratio of the actual retained face count to the original total face count ($\frac{\sum F^{\mathrm{final}}}{\sum F^{\mathrm{orig}}}$) after applying linear scaling to align with the global budget constraint. Due to the linear scaling process, the final global retention ratio closely approximates, but may not be strictly equal to, the target budget.

**Table 7:** Mesh quality comparison at three representative budget levels $r\;\in \left\{0.7,0.5,0.3\right\}$.

Method	0.7					0.5					0.3				
	HD_P99 (mm)	CDP95 (mm)	CE (mm)	WA (%)	Invalid (%)	HD_P99 (mm)	CDP95 (mm)	CE (mm)	WA (%)	Invalid (%)	HD_P99 (mm)	CDP95 (mm)	CE (mm)	WA (%)	Invalid (%)
QEM	3.91	1.62	0.009	2.73	**0**	6.87	3.88	0.014	1.73	**0**	13.13	8.25	0.027	**1.6**	**0**
ASimp	2.03	1.37	0.007	8.08	0.23	**4.58**	2.97	**0.013**	8.47	0.23	**10.72**	**7.14**	**0.027**	8.21	0.23
SABER-BIM (Ours)	**0.43**	**0.16**	**0.001**	**1.08**	**0**	7.28	**3.60**	0.019	**1.64**	**0**	11.45	7.24	0.029	2.47	0.23

Note: The definitions for HD_P99 and CDP95 are identical to those in Figure 7. CE (Mean Chamfer Error) measures the overall average deviation between the mesh surface point sets, reflecting the global geometric fidelity. WA (Non-Watertight Edge Ratio) denotes the percentage of non-watertight edges relative to the total number of edges in the simplified mesh. Invalid represents the ratio of invalid mesh faces to the total faces of the simplified mesh. For all metrics, lower values indicate better performance.

**Table 8:** Mesh quality comparison of different variants at three budget levels ($r\in \left\{0.7,0.5,0.3\right\}$).

Variant	0.7				0.5				0.3			
	HD_P99 (mm)	CDP95 (mm)	CE (mm)	Invalid (%)	HD_P99 (mm)	CDP95 (mm)	CE (mm)	Invalid (%)	HD_P99 (mm)	CDP95 (mm)	CE (mm)	Invalid (%)
baseline	1.40	0.59	0.0045	0	8.04	5.14	0.023	0	18.08	10.54	0.036	0.23
mse_only	1.48	0.66	0.0048	0	9.29	5.69	0.023	0	23.22	14.66	0.038	0.40
no_multi_scale	1.34	0.59	0.0042	0	13.65	7.90	0.023	0	25.35	16.53	0.039	0.26
no_semantic	2.33	1.23	0.0095	0	18.39	11.75	0.025	0.2	30.40	20.64	0.045	0.32
no_uncertainty	1.82	0.83	0.0045	0	19.92	12.64	0.032	0	29.95	19.93	0.043	0.32

Note: Lower is better.

**Table 9:** Budget prediction accuracy for ablation variants.

Variant	MAE	RMSE	MAPE (%)	R^2^
baseline	0.066	0.129	17.86	0.89
mse_only	0.063	0.124	14.89	0.89
no_multi_scale	0.094	0.145	20.78	0.86
no_semantic	0.296	0.437	61.41	−0.26
no_uncertainty	0.169	0.241	40.25	0.61

Note: Lower is better except for *R*^2^.

## Data Availability

The original contributions presented in this study are included in the article. Further inquiries can be directed to the corresponding author.

## References

[1] Tao F., Zhang H., Liu A., Nee A.Y.C. (2019). Digital Twin in Industry: State-of-the-Art. IEEE Trans. Ind. Inform..

[2] Latsou C., Ariansyah D., Salome L., Erkoyuncu J.A., Sibson J., Dunville J. (2024). A unified framework for digital twin development in manufacturing. Adv. Eng. Inform..

[3] Volk R., Stengel J., Schultmann F. (2014). Building Information Modeling (BIM) for existing buildings—Literature review and future needs. Autom. Constr..

[4] Machacek Z., Hercik R., Vaclavik A., Zemanek J., Hameed I.A., Koziorek J. (2025). Modern trends and industrial use cases of digital twin technology with 3D behavioral representation. J. Intell. Manuf..

[5] Huang M.Q., Zhu H.M., Ninić J., Zhang Q. (2022). Multi-LOD BIM for underground metro station: Interoperability and design-to-design enhancement. Tunn. Undergr. Space Technol..

[6] Xu Z., Zhang L., Li H., Lin Y.-H., Yin S. (2020). Combining IFC and 3D tiles to create 3D visualization for building information modeling. Autom. Constr..

[7] Building SMART International Limited (2021). Industry Foundation Classes 4.0.2.1.

[8] Zhu J., Nisbet N., Kang R., Wen Y., Wang M., Brilakis I. (2026). Revealing the internal structure of IFC-Graph for efficient querying and knowledge discovery. Adv. Eng. Inform..

[9] Zhu J., Wu P., Anumba C. (2021). A Semantics-Based Approach for Simplifying IFC Building Models to Facilitate the Use of BIM Models in GIS. Remote Sens..

[10] Garland M., Heckbert P.S. (1997). Surface simplification using quadric error metrics. Proceedings of the 24th Annual Conference on Computer Graphics and Interactive Techniques—SIGGRAPH ’97.

[11] Chang H., Dong Y., Zhang D., Su X., Yang Y., Lee I. (2023). Review of Three-Dimensional Model Simplification Algorithms Based on Quadric Error Metrics and Bibliometric Analysis by Knowledge Map. Mathematics.

[12] Lan J., Zeng B., Li S., Zhang W., Shi X. (2025). A Deep Learning-Based Salient Feature-Preserving Algorithm for Mesh Simplification. Comput. Mater. Contin..

[13] Xia W., Luo Y., Fan J., Wang S., Liu X., Zhang Y., Fei L., Wang W., Zhang B., Zhang J. (2025). Semantic-aware Multi-Scale Simplification of Urban-Scale 3D Real-Scene Mesh Models. Int. Arch. Photogramm. Remote Sens. Spat. Inf. Sci..

[14] Rossignac J., Borrel P., Falcidieno B., Kunii T.L. (1993). Multi-resolution 3D approximations for rendering complex scenes. Modeling in Computer Graphics.

[15] Turk G. (1992). Re-tiling polygonal surfaces. Proceedings of the 19th Annual Conference on Computer Graphics and Interactive Techniques.

[16] Hoppe H., Whitton M.C. (2023). Progressive Meshes. Seminal Graphics Papers: Pushing the Boundaries.

[17] Liu Z., Zhang C., Cai H., Qv W., Zhang S. (2022). A Model Simplification Algorithm for 3D Reconstruction. Remote Sens..

[18] Xie Y., Zhang Y., Hu Y., Zhan N., Sun T., Zhu J., Zhu Q. (2026). A Lightweight Approach to Railway Infrastructure BIM Models Considering Geometric Detail Features. Geomat. Inf. Sci. Wuhan Univ..

[19] Liang Y., He F., Zeng X. (2020). 3D mesh simplification with feature preservation based on whale optimization algorithm and differential evolution. Integr. Comput.-Aided Eng..

[20] Zhang Y., Wang S., Zheng Q., Zhang H. (2023). A Lightweight Algorithm for 3D Mesh Models with Preserved Detail Geometric Features. J. Comput. Appl..

[21] Qi C.R., Su H., Kaichun M., Guibas L.J. PointNet: Deep Learning on Point Sets for 3D Classification and Segmentation. Proceedings of the 2017 IEEE Conference on Computer Vision and Pattern Recognition (CVPR).

[22] Qi C.R., Yi L., Su H., Guibas L. (2017). PointNet++: Deep Hierarchical Feature Learning on Point Sets in a Metric Space. arXiv.

[23] Wang Y., Sun Y., Liu Z., Sarma S.E., Bronstein M.M., Solomon J.M. (2019). Dynamic Graph CNN for Learning on Point Clouds. ACM Trans. Graph..

[24] He Y., Yu H., Liu X., Yang Z., Sun W., Anwar S., Mian A. (2025). Deep learning based 3D segmentation in computer vision: A survey. Inf. Fusion.

[25] Guo Y., Wang H., Hu Q., Liu H., Liu L., Bennamoun M. (2021). Deep Learning for 3D Point Clouds: A Survey. IEEE Trans. Pattern Anal. Mach. Intell..

[26] Lee C.H., Varshney A., Jacobs D.W. Mesh saliency. Proceedings of the SIGGRAPH05: Special Interest Group on Computer Graphics and Interactive Techniques Conference.

[27] Luan W., Liu C., Pang H. Skeleton-bridged Mesh Saliency and Mesh Simplification. Proceedings of the 2020 4th International Conference on Computer Science and Artificial Intelligence.

[28] Feng M., Zhang L., Lin X., Gilani S.Z., Mian A. (2020). Point attention network for semantic segmentation of 3D point clouds. Pattern Recognit..

[29] Hanocka R., Hertz A., Fish N., Giryes R., Fleishman S., Cohen-Or D. (2019). MeshCNN: A network with an edge. ACM Trans. Graph..

[30] Potamias R.A., Ploumpis S., Zafeiriou S. Neural Mesh Simplification. Proceedings of the 2022 IEEE/CVF Conference on Computer Vision and Pattern Recognition (CVPR).

[31] Dovrat O., Lang I., Avidan S. S-NET: Learning to Sample. Proceedings of the 2019 IEEE/CVF Conference on Computer Vision and Pattern Recognition (CVPR).

[32] Lang I., Manor A., Avidan S. SampleNet: Differentiable Point Cloud Sampling. Proceedings of the 2020 IEEE/CVF Conference on Computer Vision and Pattern Recognition (CVPR).

[33] Liu S., Wang R., Zhan H., Yu J. (2025). TetSimNet: A tetrahedral mesh simplification network model for preserving analysis accuracy. Eng. Comput..

[34] Maruani N., Ovsjanikov M., Alliez P., Desbrun D. (2024). PoNQ: A Neural QEM-based Mesh Representation. arXiv.

[35] Lin L., Kang H., Shi Y., Duan H., El Saddik A., Cai W. ASimp: Automatic High-Poly 3D Mesh Simplification for Preprocessing Based on QoE. Proceedings of the 2025 IEEE International Conference on Multimedia and Expo (ICME).

[36] Guo M.H., Cai J.X., Liu Z.N., Mu T.-J., Martin R.R., Hu S.-M. (2021). PCT: Point cloud transformer. Comput. Vis. Media.

[37] Emunds C., Pauen N., Richter V., Frisch J., van Treeck C. (2021). IFCNet: A benchmark dataset for IFC entity classification. arXiv.

[38] Emunds C., Pauen N., Richter V., Frisch J., van Treeck C. (2022). SpaRSE-BIM: Classification of IFC-based geometry via sparse convolutional neural networks. Adv. Eng. Inform..

